# Transcatheter closure of paravalvular leak after Bentall surgery in a Marfan patient: a rare case report

**DOI:** 10.3389/fcvm.2025.1734565

**Published:** 2026-01-15

**Authors:** Shichao Guo, Zhiyuan Wang, Ning Zhang, Yanbo Dong, Huijun Zhang, Youwei Zhao

**Affiliations:** 1 Department of Cardiovascular Surgery, The First Hospital of Hebei Medical University, Shijiazhuang, Hebei, China; 2 Department of Ultrasound, The First Hospital of Hebei Medical University, Shijiazhuang, Hebei, China

**Keywords:** Bentall procedure, Marfan syndrome, paravalvular leak, pseudoaneurysm, transcatheter closure

## Abstract

Paravalvular leak (PVL) following Bentall surgery in patients with Marfan syndrome is exceedingly rare. A 38-year-old Marfan patient underwent Bentall + Sun's procedure for Stanford type A aortic dissection with severe aortic regurgitation. On the third month after surgery, the patient was readmitted due to exertional dyspnea. Echocardiography revealed a paravalvular leak with significant left-to-right shunting, leading to symptoms including exertional dyspnea, hepatomegaly, and heart failure. After adequate preparation, the leak was successfully closed using a symmetric VSD occluder. Post-procedural imaging showed near-complete resolution of the leak, significant reduction in the pseudoaneurysm size, and improvement in heart failure symptoms. The patient was discharged in stable condition.

## Introduction

The incidence of paravalvular leak after surgical aortic valve replacement ranges from 2% to 17% (1, 2). Patients with Marfan syndrome are at higher risk due to underlying connective tissue disorders (3, 4). There has been no reported case of PVL caused by valve detachment following Bentall surgery in a Marfan patient with aortic dissection. This case involves a Marfan patient with a BMI of 17.5 who developed PVL three months after Bentall surgery. Given the high risk of reoperation, transcatheter closure using a VSD occluder was carefully evaluated and successfully performed.

## Case presentation

**Figure 1:**
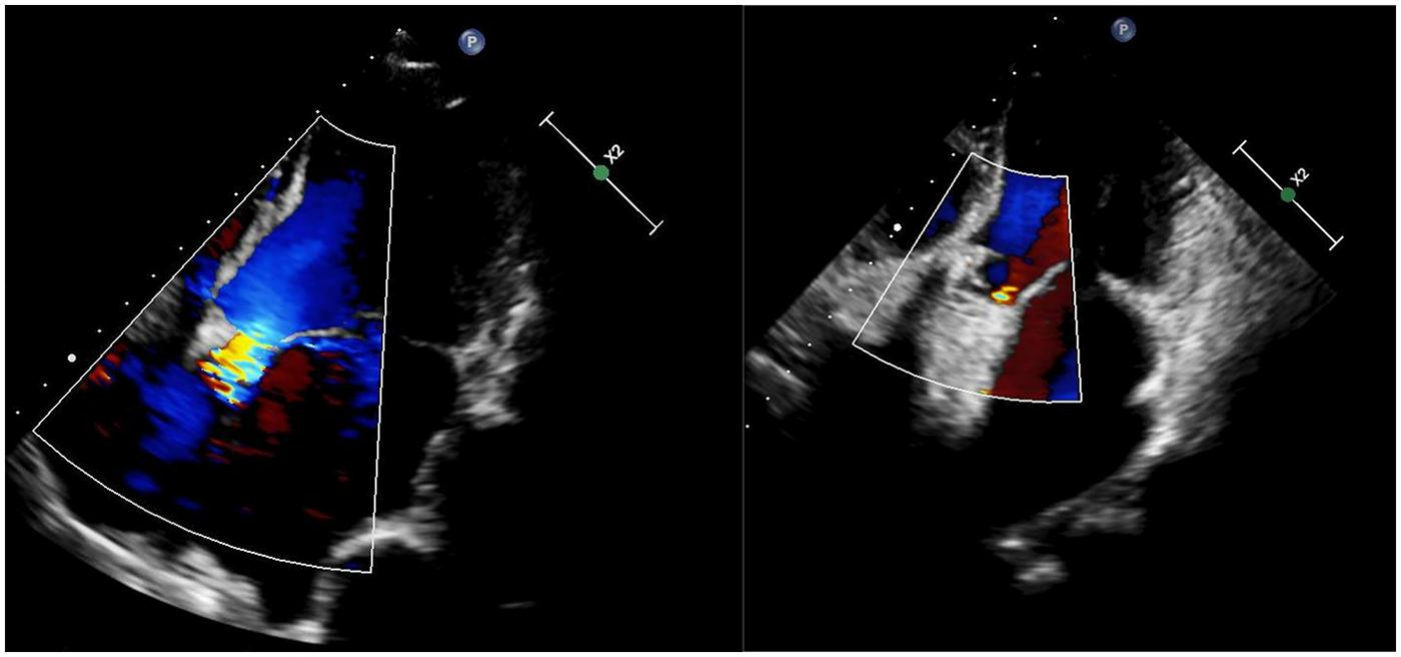
Echocardiograms before (left) and after (right) the procedure.

**Figure 2:**
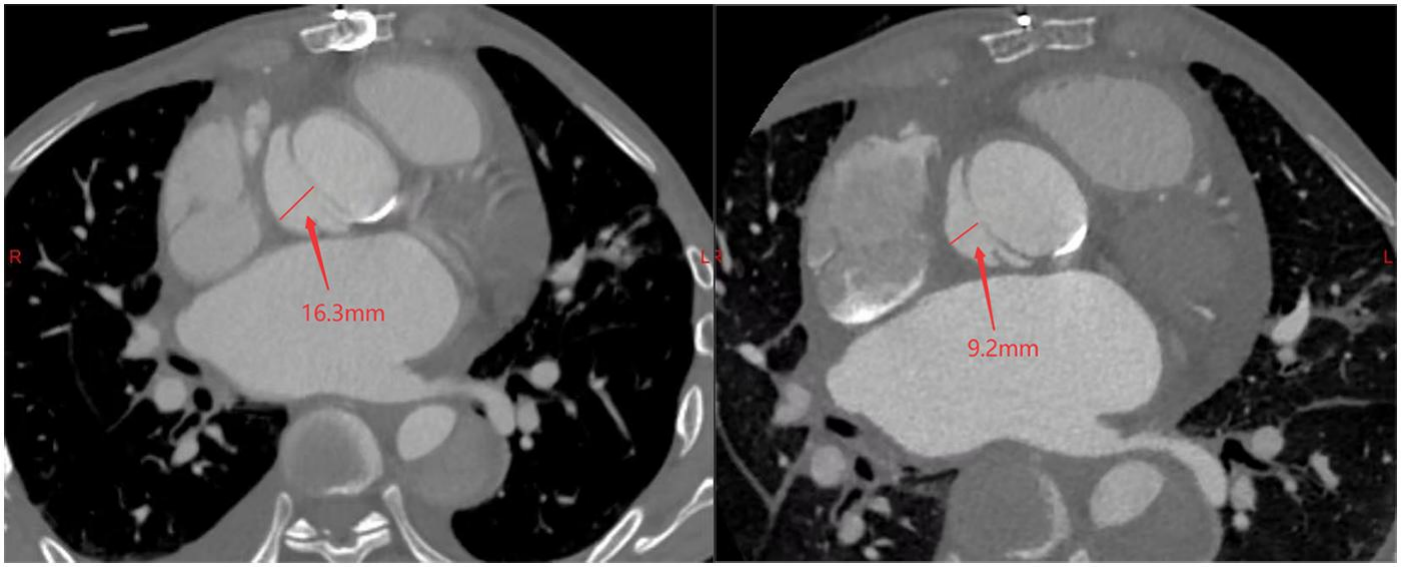
Pre-procedural (left) and post-procedural (right) CT scans demonstrating the pseudoaneurysm at its maximum width.

A 38-year-old male with Marfan syndrome underwent Bentall + Sun's surgery for aortic dissection. Three months later, he was readmitted with exertional dyspnea. Echocardiography confirmed a paravalvular leak with high-velocity flow across the defect into the right atrium via a surgically created shunt, resulting in heart failure. The patient declined reoperation. Multimodal imaging—including echocardiography (Figure 1, Supplementary Video S1) and computed tomography (Figure 2, Supplementary Video S2)—was used to plan transcatheter PVL closure.

**Figure 3:**
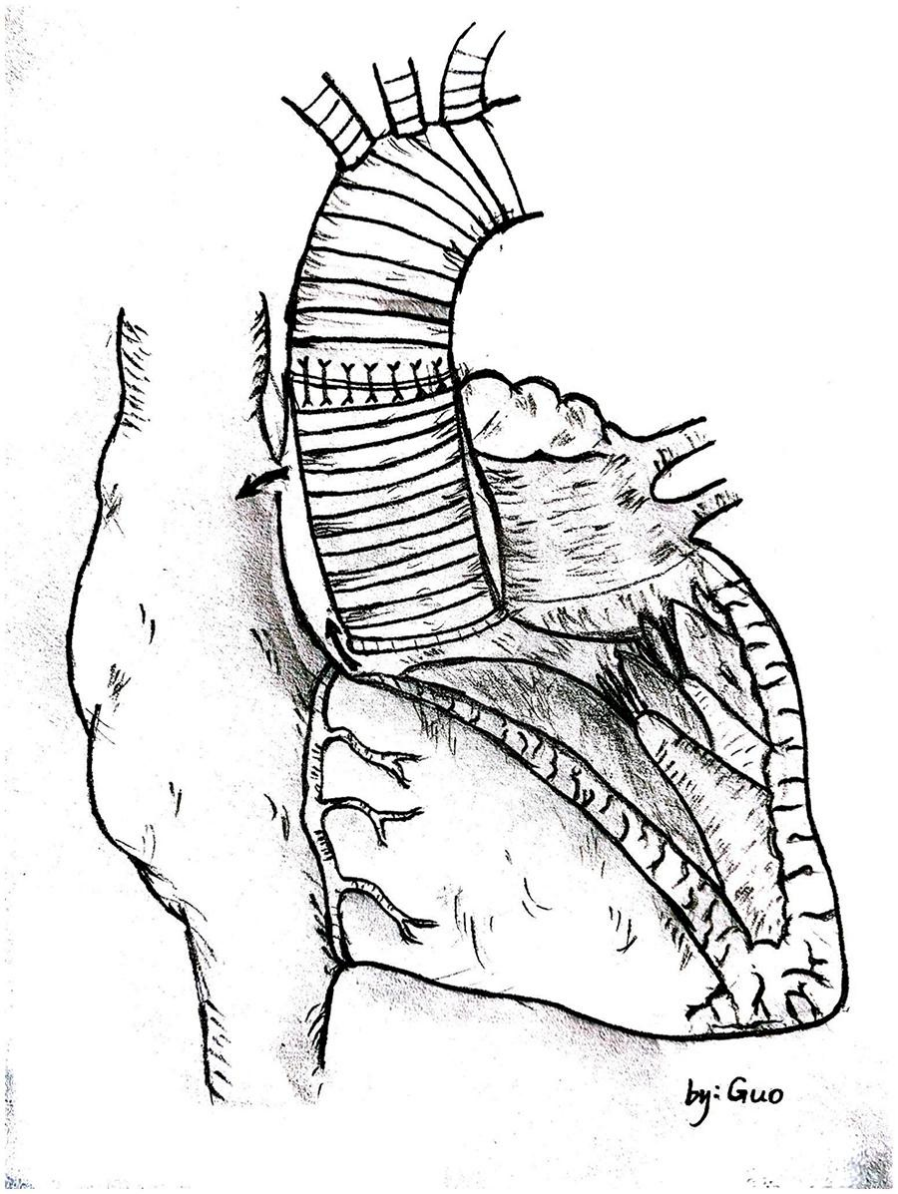
Hand-drawn diagram showing blood shunting from the paravalvular leak into the pseudoaneurysm and then to the right atrium through the cabrol shunt.

**Figure 4:**
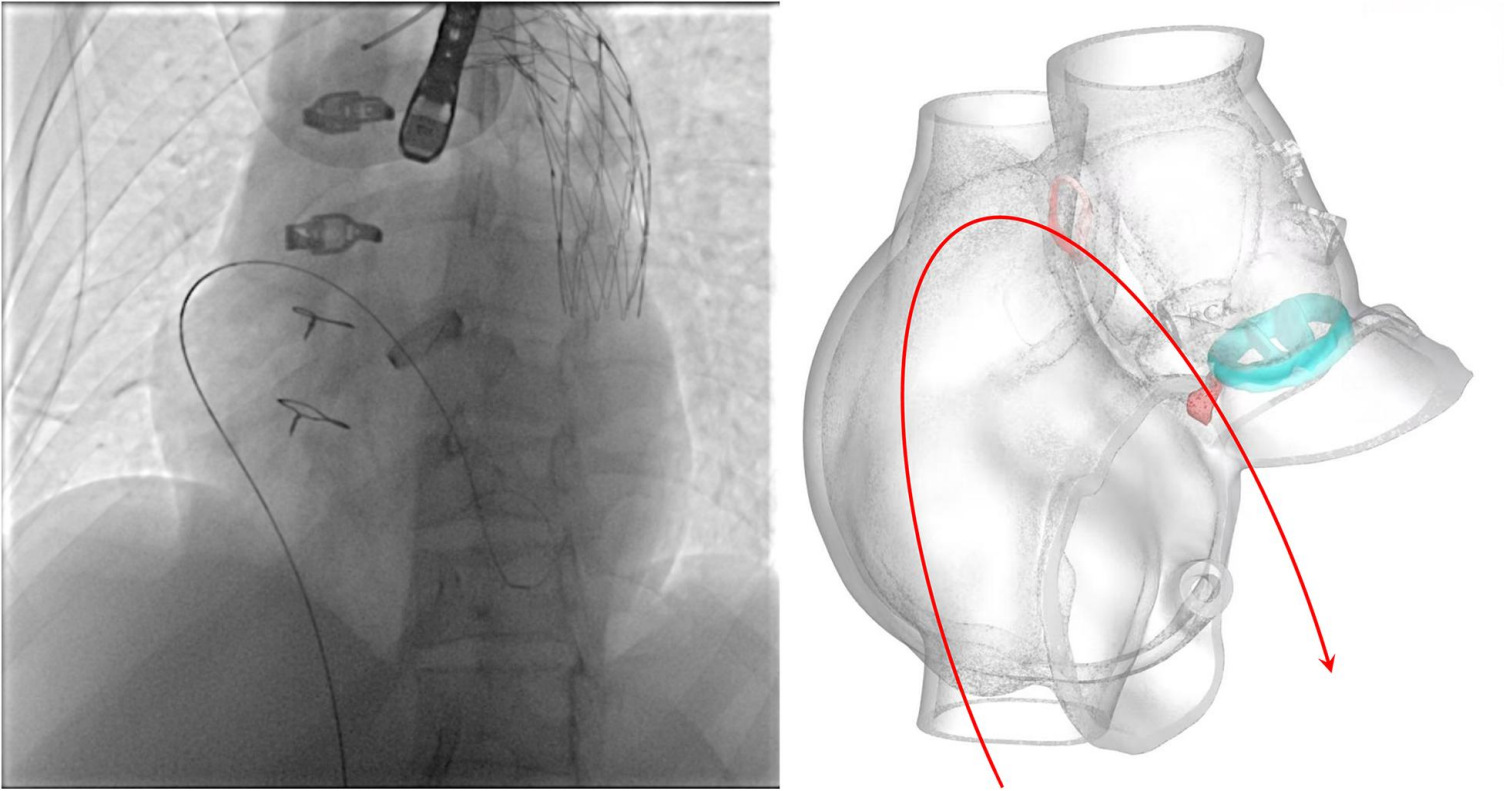
Schematic illustrating the pathway of the guidewire.

Unlike typical post-aortic valve replacement PVL, this case involved separation between the graft and native annulus (Figure 3), forming a pseudoaneurysm (A surgically-created autologous aortic adventitial chamber, communicating with the right atrium, serves to reduce surgical bleeding and is subsequently thrombosed for closure). The femoral arterial approach allowed only antegrade access to the defect. Left ventricular angiography was attempted to locate the leak, but it interfered with mechanical valve function, causing progressive bradycardia and hypotension, necessitating abandonment of the femoral approach. Subsequently, a right atrial approach was used to access the pseudoaneurysm via the Cabrol shunt from the initial surgery, followed by retrograde crossing into the left ventricle to establish a working pathway (Figure 4).

Initial attempt with a 6# VSD occluder (10 mm symmetric discs, 4 mm waist height, 6 mm waist diameter) failed due to device dislodgement. A 10# VSD occluder (14 mm symmetric discs, 4.5 mm waist height, 10 mm waist diameter) was then successfully deployed.

Post-procedural echocardiography (Figure 1, Supplementary Video S3) showed minimal residual leak. Follow-up CT (Figure 2) demonstrated significant reduction in pseudoaneurysm size. No complications—such as hemolysis, device migration, new PVL, or recurrent heart failure—were observed. The outcome was satisfactory.

At the one-month follow-up (completed after surgery), the patient was asymptomatic. Echocardiography showed significantly improved ejection fraction, normalized right heart dimensions, and only a trivial residual leak around the device. Notably, the previously observed left-to-right shunt via the Cabrol shunt was no longer detectable, indicating excellent hemodynamic results.

## Discussion

Surgical reintervention was once the definitive treatment for symptomatic moderate-to-severe PVL. However, reoperation carries significantly higher morbidity and mortality (up to 10%–15%) due to pericardial adhesions and altered anatomy (5–8). Transcatheter PVL closure has emerged as a viable alternative, receiving a Class IIa recommendation in the 2021 ESC Valvular Heart Disease Guidelines for high-risk surgical candidates (9). Studies demonstrate comparable efficacy to surgical repair, with lower mortality and shorter hospital stays (10–12).

Not all PVLs require intervention. Accepted indications for transcatheter closure include: hemolysis, symptomatic heart failure, mild-to-moderate PVL with declining left ventricular ejection fraction (LVEF), or progressive left ventricular enlargement. Relative indications include asymptomatic mild-to-moderate PVL, risk of infective endocarditis, and post-TAVI PVL (13). This patient met absolute criteria due to heart failure, moderate PVL, reduced LVEF, and left ventricular enlargement.

Route selection is critical and depends on anatomical specifics and technical feasibility. Options include retrograde femoral arterial, antegrade transseptal mitral, and transapical approaches (13, 14). Device selection must ensure stable closure without interfering with valve function or coronary flow. Commonly used devices include PDA, VSD occluders. Multiple devices may be required for complex or multifocal leaks (11, 13, 14). In this case, both the approach and device were selected under challenging constraints, yet proved successful.

Despite promising outcomes, limitations remain. PVLs often exhibit crescentic or irregular morphology, and the lack of dedicated devices may result in residual regurgitation. Hemolysis—caused by high-velocity flows shear stress—requires careful device selection. Other risks include device embolization, thromboembolism, and coronary obstruction (13).

Future advancements in 3D printing and computational modeling may improve preoperative planning and outcomes (15–17). Dedicated devices designed for PVL anatomy are also eagerly anticipated.

## Conclusion

Transcatheter closure is a safe, effective, and minimally invasive option for high-risk patients with symptomatic PVL after aortic valve replacement. Success depends on appropriate patient selection, meticulous multimodal imaging, individualized procedural planning, and collaborative decision-making within a heart team comprising cardiologists, cardiac surgeons, and imaging specialists. This case adds to the growing evidence supporting transcatheter therapy in complex scenarios.

## Data Availability

The datasets presented in this study can be found in online repositories. The names of the repository/repositories and accession number(s) can be found in the article/Supplementary Material.

## Supplementary material

Supplementary Video S1 Preoperative echocardiography in the three-chamber view shows a significant shunt across the paravalvular leak (indicated by the arrow).

Supplementary Video S2 Preoperative contrast-enhanced CT of the aortic root shows the paravalvular leak (indicated by the arrow).

Supplementary Video S3 Postoperative echocardiography in the five-chamber view shows the occluder (indicated by the arrow) with a trivial shunt across the device.
